# Ultrasonic diagnosis of delayed hematoma during emergency removal of traumatic intracranial hematoma: A case report and literature review

**DOI:** 10.1097/MD.0000000000033484

**Published:** 2022-04-07

**Authors:** Yan Weng, Ziqi Shang, Qing Zhong

**Affiliations:** a Department of Anesthesiology, the People’s Hospital of Jianyang, Jianyang, Sichuan Province, China.

**Keywords:** case report, delayed hematoma, traumatic intracranial hematoma, ultrasound diagnosis

## Abstract

It is emergency and vital during neurosurgical procedure in traumatic intracranial when an acute intraoperative brain bulge (AIBB) is occurred. It is important to get a diagnosis quickly.

**Patient concerns:**

A 44-year-old man was undergone a neurosurgical procedure for the left side of traumatic intracranial hematoma. An AIBB was occurred during the surgery. Computed tomography (CT) is always used in diagnosis when an AIBB is occurred, but more time is needed when CT is conducted.

**Diagnoses:**

We diagnosed the AIBB through bedside real-time ultrasound, and a delayed hematoma which caused the AIBB was found.

**Interventions:**

A further neurosurgical procedure of right intracranial hematoma was performed for the patient.

**Outcomes:**

The surgical effect and the patient’s prognosis were significantly improved.

**Lessons:**

Through this patient, we should pay more attention to the application of perioperative of real-time ultrasonic monitoring, to provide more convenience for surgical patients, and improve the prognosis of them.

## 1. Introduction

Traumatic intracranial hematoma is a common disease among car accident injuries. Removal of intracranial hematoma is the most common therapy in traumatic intracranial hematoma. Acute intraoperative brain bulge (AIBB), which is caused by high intracranial pressure (ICP),^[1]^ is a severe condition during the surgery. AIBB would seriously affect the prognosis of patients, even more it always leads to high disability rate and fatality rate. It is necessary to identify the cause as soon as possible and take appropriate treatment measures to save the lives of patients. Ultrasound is currently taking off in the medical world and is widely used in various fields of anesthesiology. In this case, the application of ultrasonic diagnosis in AIBB was studied. The bedside ultrasonic diagnosis, which improved the prognosis of the patient, played an important role in the operation plan during the surgery.

## 2. Case presentation

A 44-years-old man was admitted to the emergency for “headache, bilateral external auditory canal bleeding, nausea and vomiting for 1 + hours after falling from a motorcycle.”

He presented with headache, accompanied by bilateral external auditory canal bleeding, with nausea and vomiting. In the form of non-ejection, he vomited several times with dark brown stomach contents but the specific amount is unknown. It was unknown whether he had a history of coma, and there were no convulsions and incontinence.

Physical examination when admitted to the intensive care unit: the patient was lethargic, with a pulse of 89 beats per min (bpm), a respiration of 16 bpm, a blood pressure of 97/60 mm Hg (1 mm Hg = 0.133 kPa), and a pulse oxygen saturation (SpO_2_) of 100%. The pupils were equal in size and round, with a diameter of about 3 mm, and the light reflex exists, the eye movement was sufficient, and bloody fluid could be seen flowing out of the bilateral external auditory canals. There was blood in the nasal cavity bilaterally with no active bleeding. There was tenderness in the middle part of the right clavicle, and a bone rubbing sensation was palpable. Chest and abdominal examination showed no positive signs. Skin contusion and laceration can be seen in the big toe of the left foot. The limbs can be moved simply as instructed. There is no obvious percussion pain in the axial direction. Limb muscle strength grade V with low tension. He uncooperated with sensory examination. And bilateral knee tendon reflex symmetry normal, bilateral Babinski sign negative. The Glasgow Coma Scale score was 12 (E3V5M4 Coma Scale: The patient would open his eyes when he was called out, his orientation was normal, and his limbs would contract when painful stimuli).

**Figure 1.**
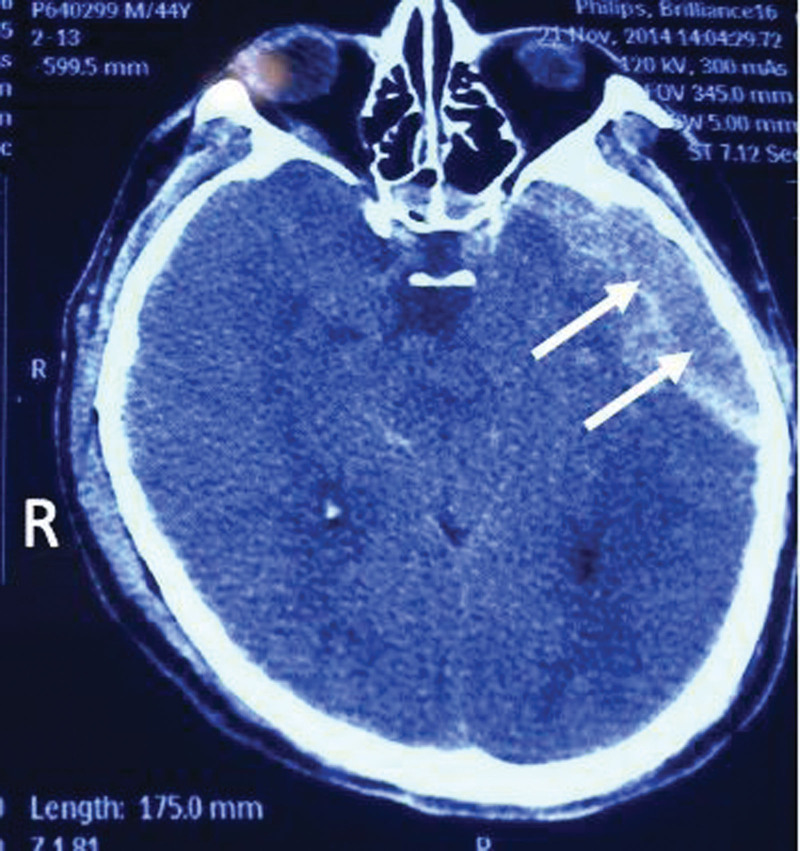
Mixed subdural and epidural hematoma in the left frontotemporal region is highly likely, with brainstem compression.

Head computed tomography (CT) in outpatient and emergency department of our hospital at the time of admission (e.g., Fig. 1).

The main admission diagnosis: traumatic subdural hematoma, traumatic epidural hematoma.

Emergency evacuation of the left intracranial hematoma was planned. Under the general anesthesia and tracheal intubation, the operation went well, about 30 mL accumulated blood was removed. The patient’s brain tissue continued to bulge during the process of removing the hematoma, and the operation space was lost, the bleeding in the operation area was obvious, and hemostasis was difficult. AIBB was occurred.

**Figure 2.**
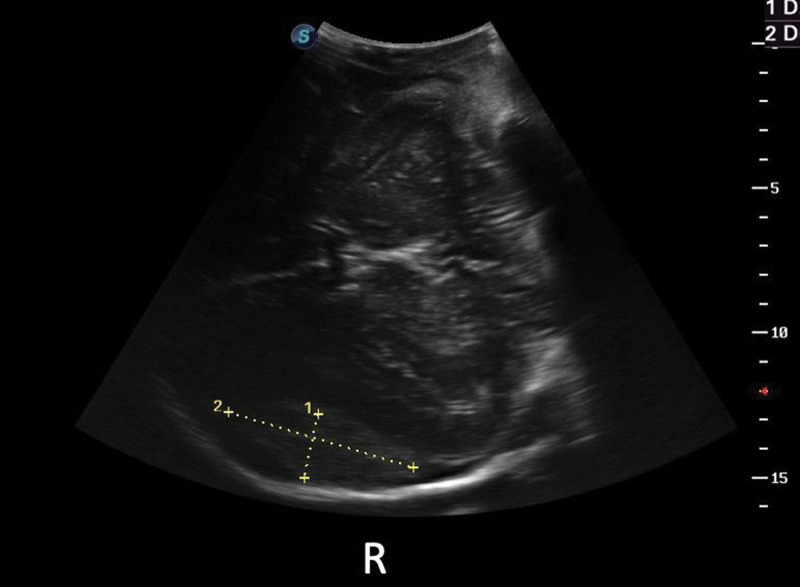
A 2.3 cm × 6.5 cm low-medium echogenic homogeneous fusiform mass with clear borders in the right temporal part was found.

In order to find the cause of AIBB, a bedside ultrasound exploration was performed by the anesthesiologist. The S8EXP (Huiying) 2 to 5 MHz convex array probe was choosed, which was wraped with a sterile endoscope sleeve. Then he placed the probe on the open brain tissue surface where the bone flap was removed on the left side, and a 2.3 cm × 6.5 cm low-medium echogenic homogeneous fusiform mass with clear borders in the right temporal part was found (e.g., Fig. 2), which was significantly enlarged compared with the preoperative CT scan.

**Figure 3.**
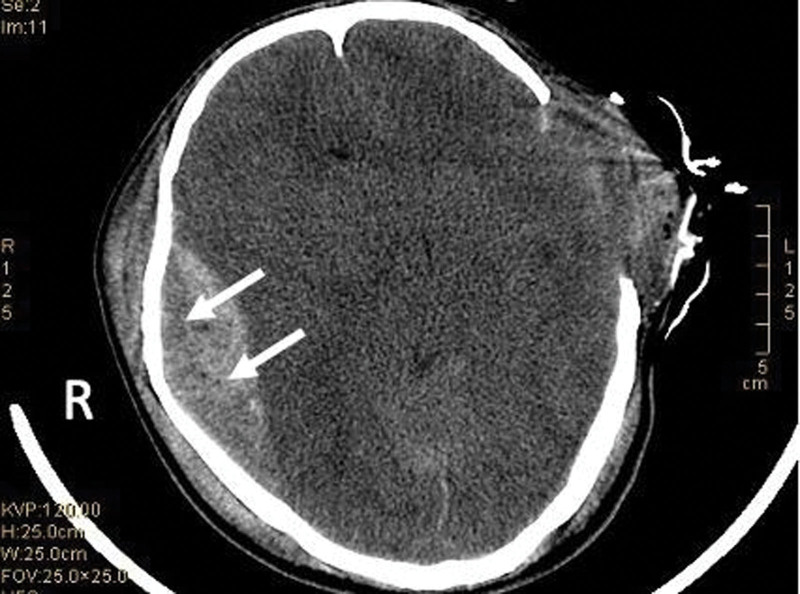
The lack of bone in the left temporal bone showed postoperative changes, and the brain tissue in the temporal lobe bulged; there were 2 epidural hematomas on the top of the right temporal bone, with a large bleeding volume of about 67 mL, cerebral herniation, obstructive hydrocephalus, and brainstem compression; multiple fractures of the right temporal bone, involving the middle ear mastoid and external auditory canal, bilateral frontotemporal soft tissue swelling.

A head CT was completed, which showed there were 2 epidural hematomas on the top of the right temporal bone, with a large bleeding volume of about 67 mL (e.g., Fig. 3).

A further surgery of right intracranial hematoma was performed, and a total of about 70 mL of epidural hematoma was removed. After the operation, his vital signs were basically stable, and he was sent to the intensive care unit with a tracheal tube for further treatment.

**Figure 4.**
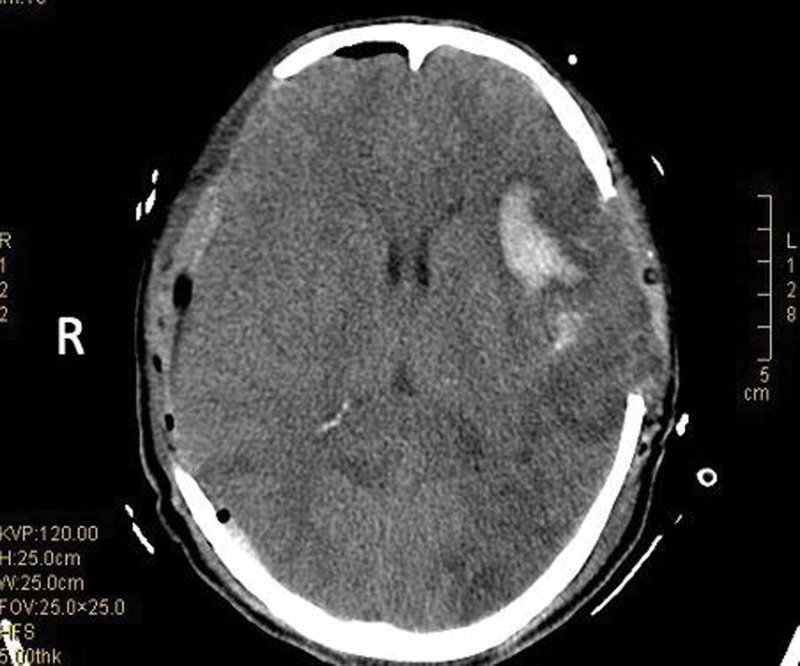
Postoperative changes in bilateral frontal, parietal, and temporal bones, swelling in both cerebral hemispheres, patchy and small patchy high-density hemorrhage in the right temporal lobe, left frontal lobe, and temporal lobe, and left frontal lobe Large flakes of low-density edema were seen in the, parietal, temporal lobes, right temporal lobes, and bilateral occipital lobes, and a little strip-like and bubbly air accumulation under the inner plate of the frontal bone and right occipital bone.

**Figure 5.**
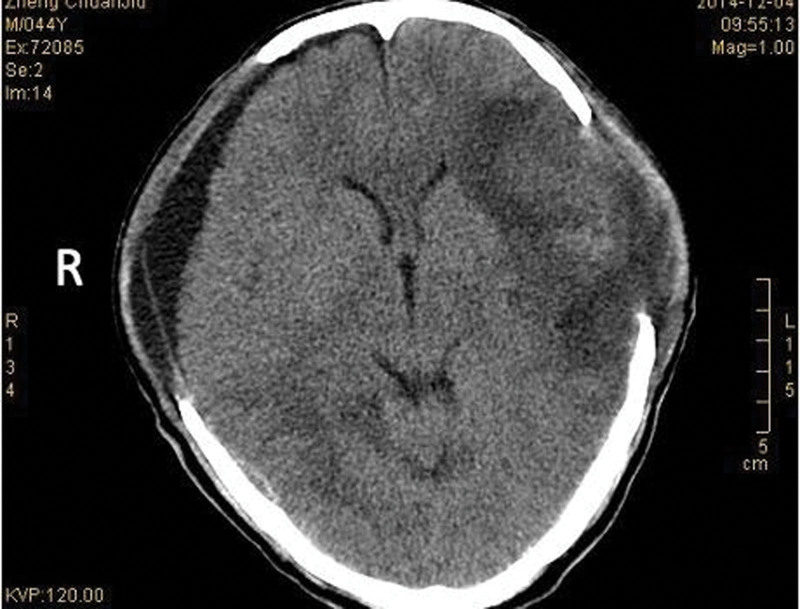
Compared with postoperative films: bilateral frontotemporal epidural hematomas had been cleared, and the edema in the operation area was better than before. Left frontotemporal lobe, the density of the residual hemorrhage in the right parietal lobe became lighter, and the partial swelling of the left frontotemporal lobe brain tissue was relieved; obstructive hydrocephalus, brain herniation, and brainstem compression had been significantly relieved.

Reexamination of the head CT at the time of 12 hours (e.g., Fig. 4), on the 13th day (e.g., Fig. 5) after the operation showed a change of better recovery. On the 17th day after the operation, the patient’s vital signs were stable, the pupils were equal in size and circle, the diameter was about 2 mm, and the light reflex was slow. The Glasgow Coma Scale score is 10 points (E4V2M4 coma scoring method: the patient could open his eyes automatically, could make a sound but cannot speak words, and the patient’s limbs could contract when the pain was stimulated). He was transferred to the neurosurgery ward to continue rehabilitation.

## 3. Discussion

Traumatic brain injury (TBI) as a serious public health problem, which afflicts roughly 55 million people worldwide and has a cost of at least US$400 billion per year.^[2]^ Because of the limited volume of the cranial cavity, the ICP will increase as a result of hemorrhage, cerebral edema, and hydrocephalus in TBI. Decompressive craniectomy has long been part of the neurosurgeon’s armamentarium for treating ICP elevation resulting from TBI.^[3]^ A phenomenon called AIBB is common during or after removal the hematoma in decompressive craniectomy. AIBB can progress rapidly and cause ischemic necrosis of brain tissue or even death.^[4]^ It is serious and difficult to deal with in moderate-to-severe TBI when occurred. And it will lead to high disability rate and fatality rate. Several reasons such as late-onset intracranial hematoma, acute diffuse brain swelling, and venous reflux disorder, were considered as mechanisms of AIBB.^[5]^ If the cause of AIBB in a given patient is late-onset intracranial hematoma, clinicians should promptly perform surgery to remove the hematoma and relieve circulation disorders, thus preventing more serious complications.^[4]^

In this case, after removing the left epidural and subdural hematoma, AIBB was occurred, with normal brain beat, and the brain tissue was soft, the venous congestion on the brain surface was light. Combined with the patient’s injury history, preoperative CT findings, and intraoperative findings, we speculated that the patient was most likely to develop late-onset intracranial hematoma, and that the risk factors might be related to hyperventilation, diuretic dehydration, and especially rapid surgical decompression.

As early as 1981, Dohrmann and Rubin^[6]^ have reported the application of ultrasound in neurosurgical operations, they found that real-time ultrasound scanning is of great use in the neurosurgical operating room for localizing small lesions within the substance of the brain, such as tumors, abscesses, cysts and so on. Because of its affordability, handiness, multimodal real-time nature, and overall by its image spatial and temporal resolution, intraoperative ultrasound is increasingly used in current neurosurgical operations.^[7]^

In the last decade, with the increasing popularity of portable ultrasound in anesthesia and critical care medicine,^[8]^ non-imaging clinician can integrate it into the clinical practice of their specialty in a short time, and ultrasound become an essential “visual instrument” in their daily clinical work. And ultrasound has been widely used in anesthesiology,^[9]^ the anesthesiologists have become proficient in the use of ultrasound, such as all kinds of nerve blocks.^[10,11]^ When AIBB occurred during the operation, the anesthesiologist preliminarily identified the cause which was late-onset intracranial hematoma, through ultrasound with the basis of intracranial ultrasound. For the safety of the patient, a head CT was completed in the imaging department, then an active surgical treatment was underwent. In the whole process of diagnosis and treatment, the anesthesiologist’s initial diagnosis of AIBB by bedside ultrasound saved the patient’s visit time. The surgical effect and the patient’s prognosis were significantly improved.

In conclusion, compared with bedside CT, bedside ultrasound is economical and easy to obtain, and bedside ultrasound has better application value for intraoperative examination and guidance of neurosurgery, and it has many advantages such as affordability, handiness, multimodal real-time nature and so on. Routine monitoring during operation needs further in-depth study.

## Author contributions

**Conceptualization:** Qing Zhong.

**Funding acquisition:** Qing Zhong.

**Investigation:** Ziqi Shang.

**Methodology:** Yan Weng.

**Writing – original draft:** Yan Weng.

**Writing – review & editing:** Yan Weng.
